# Neural responses in retrosplenial cortex associated with environmental alterations

**DOI:** 10.1016/j.isci.2021.103377

**Published:** 2021-10-28

**Authors:** Lucas C. Carstensen, Andrew S. Alexander, G. William Chapman, Aubrey J. Lee, Michael E. Hasselmo

**Affiliations:** 1 Center for Systems Neuroscience, Boston University, 610 Commonwealth Avenue, Boston, MA 02215, USA; 2 Department of Psychological and Brain Sciences, Boston University, 610 Commonwealth Avenue, Boston, MA 02215, USA; 3 Graduate Program for Neuroscience, Boston University, 610 Commonwealth Avenue, Boston, MA 02215, USA

**Keywords:** Animal physiology, Neuroscience, Behavioral neuroscience, Cellular neuroscience

## Abstract

The retrosplenial cortex (RSC) is an area interconnected with regions of the brain that display spatial correlates. Neurons in connected regions may encode an animal’s position in the environment and location or proximity to objects or boundaries. RSC has also been shown to be important for spatial memory, such as tracking distance from and between landmarks, contextual information, and orientation within an environment. For these reasons, it is important to determine how neurons in RSC represent cues such as objects or boundaries and their relationship to the environment. In the current work, we performed electrophysiological recordings in RSC, whereas rats foraged in arenas that could contain an object or in which the environment was altered. We report RSC neurons display changes in mean firing rate responding to alterations of the environment. These alterations include the arena rotating, changing size or shape, or an object being introduced into the arena.

## Highlights

• Insertion of an object induces a change in mean firing rate in retrosplenial neurons
• Cells that change mean firing rate (MFR) are not driven by speed modulation
• Population representation changes over time, but not in cells with MFR changes
• Manipulation of environmental features induces a change in mean firing rate

## Graphical abstract

**Figure:**
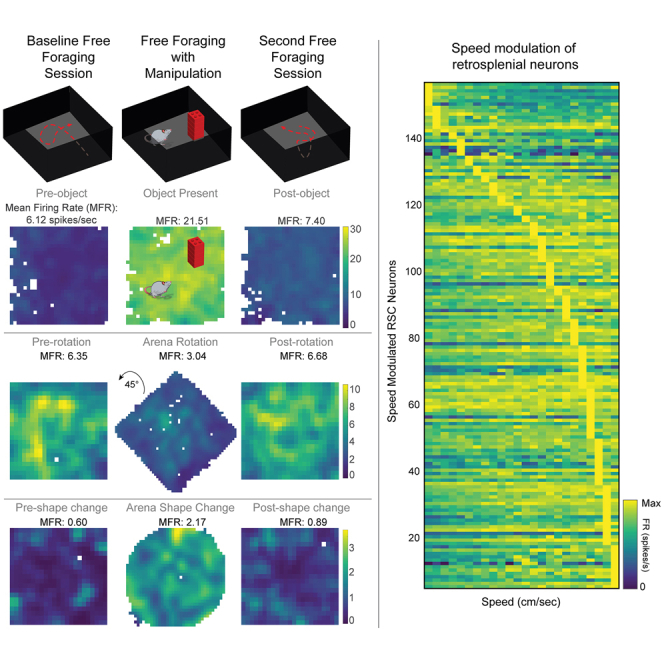

## Introduction

The retrosplenial cortex plays an important role in spatial behavior dependent upon orientation to the elements of the current spatial environment, and relationships between different coordinate frames. For example, in the human literature, damage to the retrosplenial cortex has been shown to cause impairments of orientation, including difficulty in determining the direction between landmarks in a spatial environment (Katayama et al., 1999). Functional magnetic resonance imaging has shown activation of retrosplenial cortex during tasks requiring memory of spatial views, spatial locations, spatial goals, and landmarks within a larger spatial environment(Auger et al., 2012; Chrastil et al., 2015; Epstein et al., 2007; Marchette et al., 2015; Sherrill et al., 2013). In addition, RSC is more active when viewing scenes whose spatial surroundings are known than when viewing scenes whose spatial surroundings are unknown (Epstein et al., 2007; Patai et al., 2019). Computational models have proposed that circuits in retrosplenial cortex mediate transformations between egocentric and allocentric reference frames using current head direction to map between egocentric spatial viewpoints of boundaries and an allocentric map of boundary location (Bicanski and Burgess, 2018; Byrne et al., 2007). These data and models suggest a role of the retrosplenial cortex in coding the relationship of environmental features to different reference frames.

Electrophysiological data provides some insights into the neural activity within the retrosplenial cortex involved in coding of spatial dimensions such as direction, object location, egocentric spatial viewpoints, and allocentric spatial location. For example, retrosplenial neurons code the egocentric turning direction at different points along a linear track, with responses that also depend upon broader scale coordinates such as position along the route or allocentric spatial location within a larger room (Alexander and Nitz, 2015, 2017) and has been shown to be necessary for using visual distal cues to solve spatial tasks(Hindley et al., 2014). More recently, neurons in retrosplenial cortex have been shown to respond to the egocentric position of barriers (Alexander et al., 2020; van Wijngaarden et al., 2020) similar to egocentric coding of barriers in postrhinal cortex, lateral entorhinal cortex, and dorsal striatum (Gofman et al., 2019; Hinman et al., 2019; LaChance et al., 2019; Wang et al., 2018) and allocentric coding of barriers in entorhinal cortex (Solstad et al., 2008) and subiculum (Lever et al., 2010; Poulter et al., 2021). Some neurons in the retrosplenial cortex have been shown to exhibit broad responses to changes in environmental context such as a change in color of barriers, auditory cues, and odors (Miller et al., 2021), as well as responses to goal locations (Miller et al., 2019).

Spatial behavior also requires knowledge of the location and egocentric direction to individual objects within the environment. Neurophysiological studies have shown coding of object location in the form of neural responses to the relative position of all objects in an environment found in lateral entorhinal cortex (Deshmukh and Knierim, 2011) and individual cells in hippocampus known as landmark-vector cells were shown to respond at a specific distance and direction from landmarks (Deshmukh and Knierim, 2013). Recordings in the lateral entorhinal cortex show responses to the prior location of objects (Tsao et al., 2013) as well as the direction and distance of current objects (Høydal et al., 2019). Trace responses to the prior location of an object were also observed in anterior cingulate cortex (Weible et al., 2012) and anterior retrosplenial cortex has been shown to be necessary for long-term object recognition memory(de Landeta et al., 2020; Haijima and Ichitani, 2012).

The role of retrosplenial cortex in coding the memory of landmarks within environments (Chrastil et al., 2015; Epstein et al., 2007; Marchette et al., 2015; Sherrill et al., 2013) suggests there might be changes in neural activity in this region associated with the presence of objects. Tetrode recordings of individual neurons in the retrosplenial cortex were performed before, during, and after insertions of objects into a spatial open field as rats foraged for scattered food. Retrosplenial neurons showed broad based changes in mean firing rate associated with insertion of objects. Similar changes in mean firing rate were also observed during broader alterations of environment orientation and geometry.

## Results

### Retrosplenial cortex recordings during free exploration

**Figure 1:**
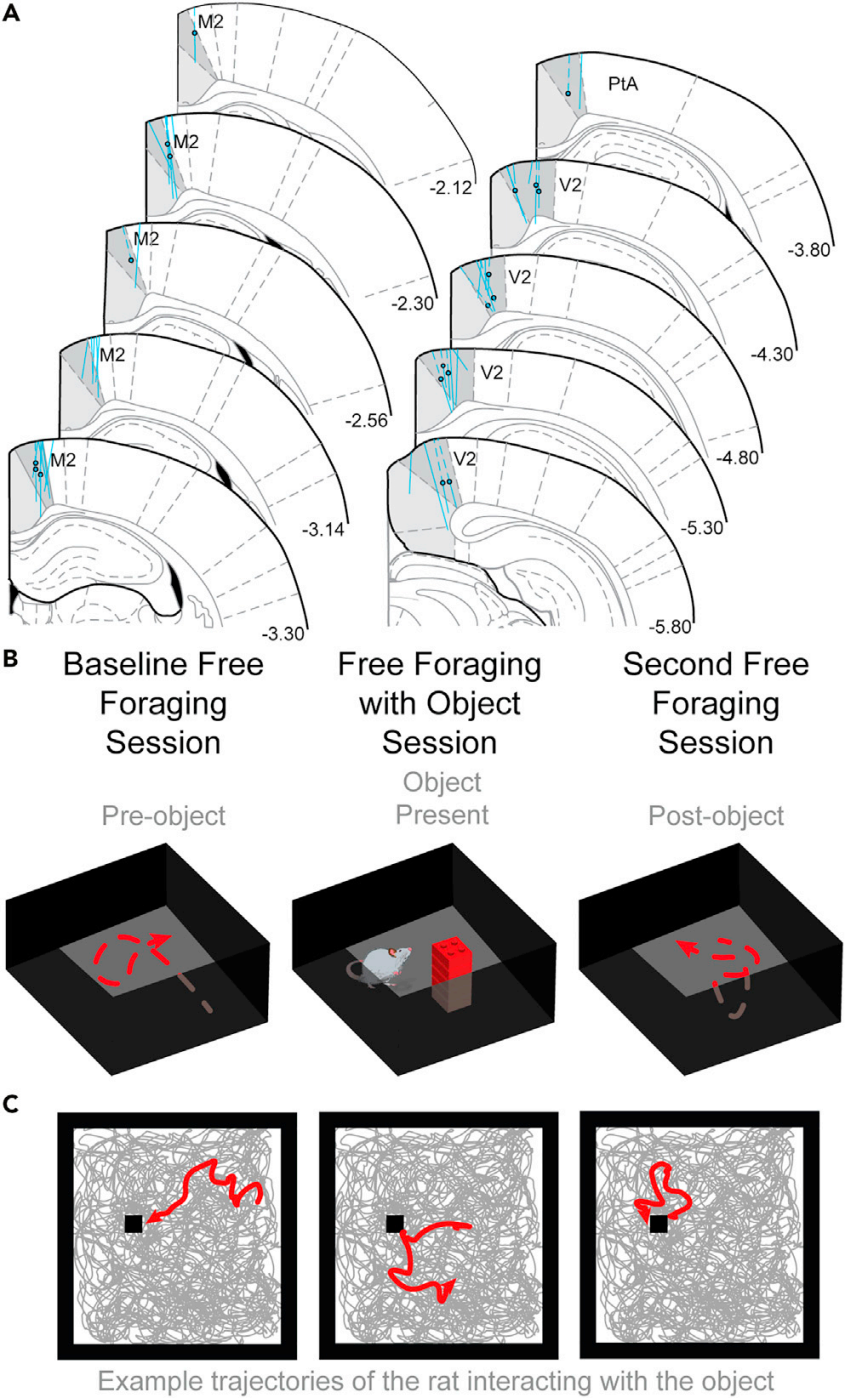
Retrosplenial cortex recordings during free exploration (A) Dashed lines indicate locations of RSC tetrode tracts where neurons with significant changes in MFR were observed. Circles indicate the most ventral location in which a neuron with a significant change in MFR was recorded. (B) The task (top) involved a minimum duration of 12 min and 45 s of baseline foraging, foraging after object insertion, and foraging after object removal. (C) Example trajectories (bottom) of the entire session shown in gray. Five second trajectory epochs of the rat near the inserted object are shown in red, and the object is shown as a black square. See also Figure S1.

We recorded 310 neurons extracellularly in the retrosplenial cortex (RSC) in either hemisphere from male or female Long-Evans rats (male = 5, female = 2) during free exploration (Figure 1A). For all sessions, rats foraged for scattered reward in 1.25m^2^ square arenas with observable fixed distal cues for a minimum duration of 12 min and 45 s (765 s) then returned to their home cage for an inter-session interval of at least one hour (Figure 1B). For object sessions, rats were returned to the same arena to forage, but an object consisting of a small tower of Lego bricks was introduced into the environment after the first baseline session. The rat was then returned to its home cage and the object was removed from the arena. After at least one hour, the rat was returned to the arena and allowed to forage in an identical manner to the first session.

### Insertion of an object induces a systematic change in mean firing rate

**Figure 2:**
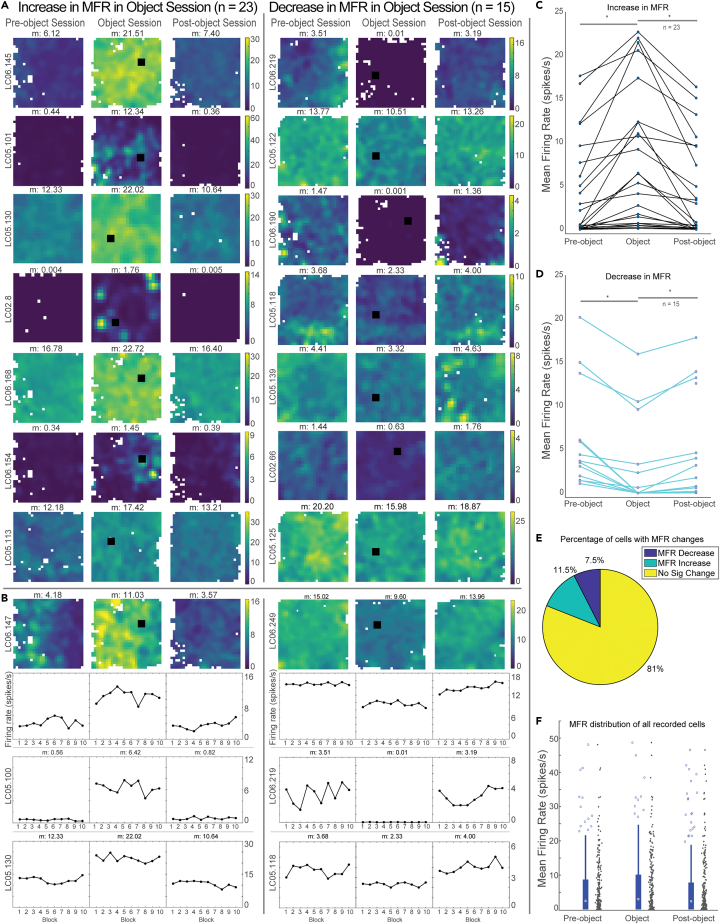
Insertion of an object induces a change in mean firing rate (A) Cells that exhibited a significant change in mean firing rate (n = 38/200, 19%) are shown. Sessions in which the mean firing rate (MFR) significantly increased during the Object session compared to both pre- and post-object (n = 23) are shown on the left. Sessions in which the MFR significantly decreased during the Object session compared to pre- and post-object (n = 15) are shown on the right. The color axis is shown at the right of each set of three ratemaps for visualization, but the true mean firing rate is shown above each ratemap. Ratemaps were normalized to the 95th percentile of the maximum firing rate of all of the 3 sessions. (B) Sessions were split into ten bins of 76.5 s and the MFR was calculated for each bin. Line plots show example neurons depicting the change in MFR across the entire session, with the middle session increasing (left) or decreasing (right) in MFR relative to the Pre- and Post-object sessions. (C) Cells that exhibited a significant increase in MFR (n = 23/38, 60.5%) are shown separated by session. (D) Cells that exhibited a significant decrease in MFR (n = 15/38, 39.5%) are shown separated by session. (E) Percentages of cells that do and do not have significant MFR changes. (F) MFR of all recorded cells separated by session is shown, with box plots indicating 25th, 50th, and 75th percentiles. Whiskers indicate the maximum and minimum MFR not considered outliers.

The most striking neurophysiological phenotype reflecting object insertions was an apparent alteration to overall firing rate (Figure 2A). To quantify the observation, each session was divided into ten blocks of equal time, and the mean firing rate (MFR) of each block was calculated as well as the mean firing rate across the entire session. The mean firing rates of all blocks of each session were compared using a Kruskal-Wallis test. The change in mean firing rate was considered significant if the MFR of all blocks within the object session was significantly different than the blocks from the other two sessions (i.e., pre and post). The mean firing rate was significantly and systematically modulated as a function of the presence or absence of an object in a large number of neurons (19%) recorded in all of the three sessions (Figures 2A–2D, n = 38/200). In 60.5% of these neurons, the mean firing rate significantly increased in the object session compared to the pre- and post-object sessions (Figure 2A, left, n = 23/38). Conversely, 39.5% of these neurons, the mean firing rate significantly decreased in the object session compared to the pre- and post-object sessions (Figure 2A, right, n = 15/38). There was a continuum of MFR increase or decrease in cells that exhibited significant changes in the object session. The mean increase in MFR was 4.0 spikes/sec (Figure 2C), and the mean decrease in MFR was 5.7 spikes/sec (Figure 2D). Out of all neurons recorded, 11.5% neurons increased and 7.5% decreased their MFR in the object session (Figure 2E, n = 23/200, n = 15/200, resp.). There were no differences between sexes in the number of cells that systematically changed their mean firing rate in the object session, though there is not sufficient statistical power to conclusively show significance.

### Cells that systematically change mean firing rate are not driven by speed modulation

**Figure 3:**
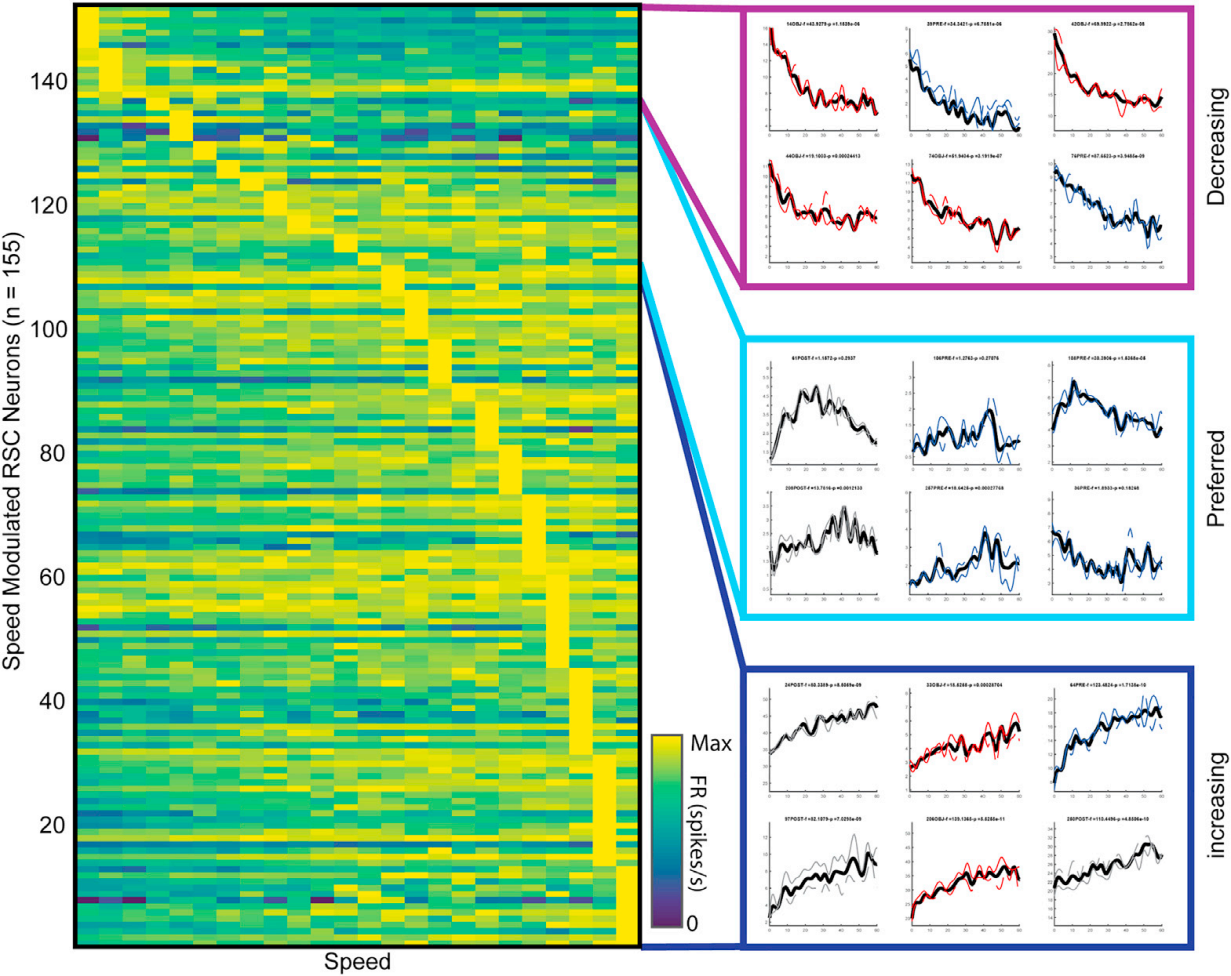
Speed modulation of retrosplenial neurons Retrosplenial cells show a spectrum of speed modulation responses. Each row shows the speed modulated firing rate across speed bins from 0 to 60 cm/s in bins of 2.5 cm/s. All three sessions were merged for visualization. Cells were sorted by the speed at which their modulated firing rate was highest. Cells were separated into increasing, preferred, and decreasing categories based on speed tuning curve shape. The black curve shows the entire session speed tuning curve, the solid gray line shows the speed tuning curve for the first half of the session, and the gray dotted line shows the speed tuning curve for the second half of the session. See also Figure S2.

A large proportion of retrosplenial neurons have been shown to be modulated by speed in electrophysiological studies in the retrosplenial cortex (Alexander et al., 2020). Accordingly, we determined cells that were speed sensitive in any one of the three sessions to determine if this influenced the change in MFR. Only 28.9% of all cells that significantly changed MFR in the object session had significant speed tuning (n = 11/38). In contrast, 77.5% of cells in the overall population showed significant speed tuning in at least 1 session (Figure 3, n = 155/200). This led us to conclude that MFR alterations were not related to systematic changes in linear speed tuning between sessions. Further, we show that there are differing speed tuning preferences in the cells that are speed modulated. Cells exhibited preferential speed tuning to a broad range of speeds, which is shown by the distribution of cells and speed tuning curves having maximum speed modulation of firing rate across all speed bins shown (Figures 3 and S2). We have qualitatively separated these speed tuning curves into three categories for additional detail, which are: “decreasing,” having a speed tuning curve that shows highest speed modulation at low speeds; “preferred,” showing a firing rate that reaches a maximum at a specific preferred speed range; and “increasing,” which show increasing speed modulation as speed increases.

### Mean firing rate population vector correlation analyses

**Figure 4:**
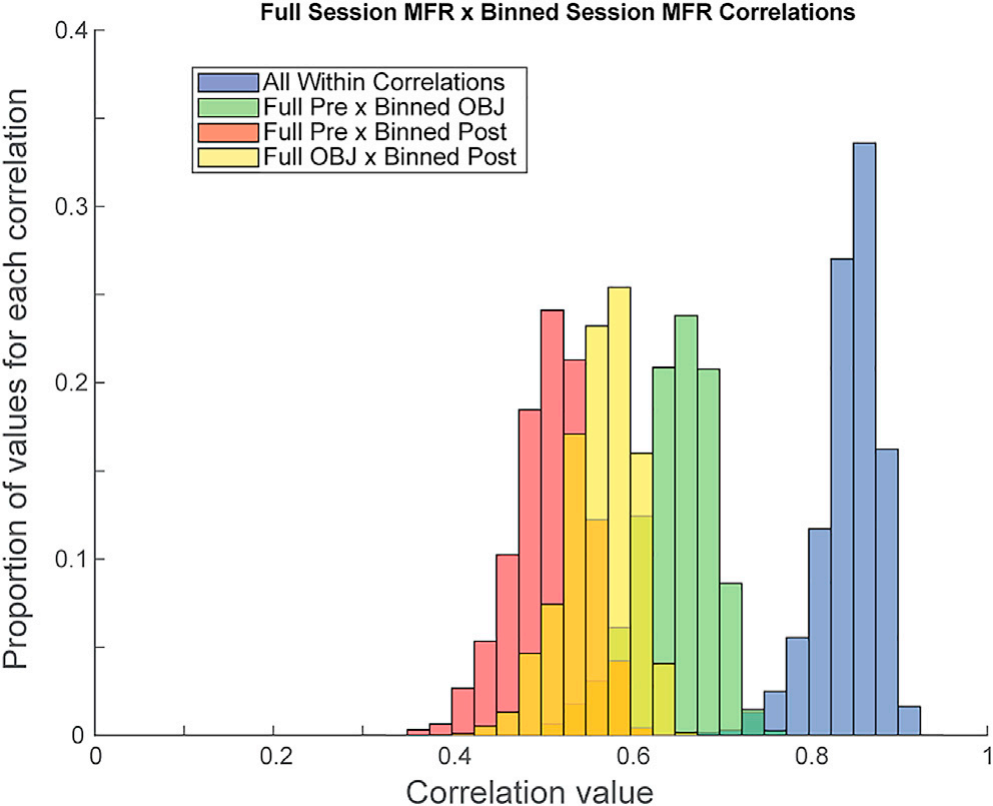
Mean firing rate population vector correlation analyses Histograms represent the correlations between population MFR vectors for each session, correlated with each bin of each session's MFR vector in 400 ms bins. Blue histogram indicates all correlations between sessions of the same type (e.g. Pre-object population vector & binned Pre-object vector). Green histogram indicates correlations between Pre-object sessions and Object sessions. Red histogram indicates correlations between Pre- and Post-object sessions. Yellow histogram indicates correlations between Object and Post-object sessions.

**Figure 5:**
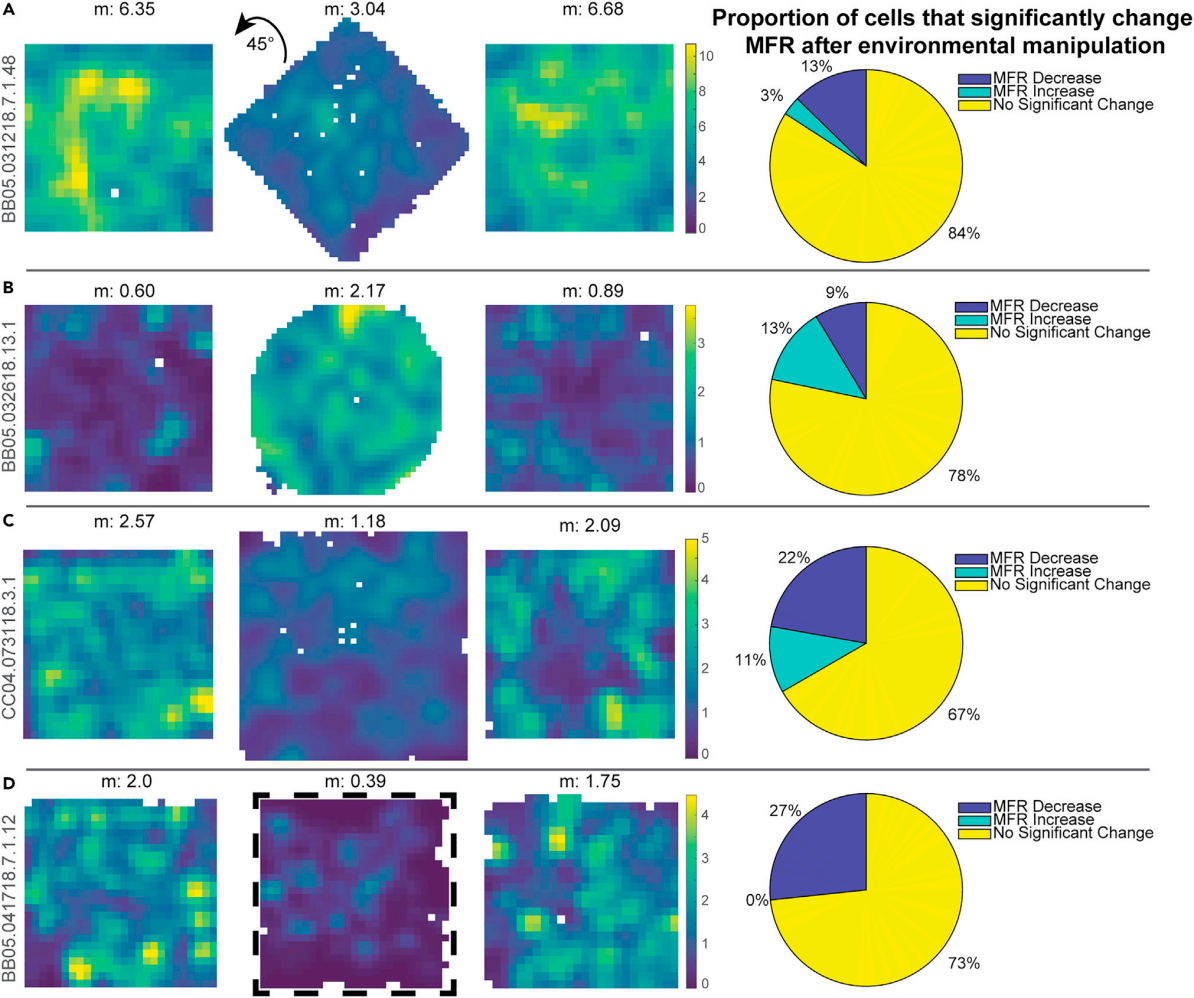
Manipulation of environmental features induces a change in mean firing rate (A) 8/63 (12.7%) cells significantly decreased their MFR when the arena was rotated by 45°, while 2/63 (3.2%) cells significantly increased their MFR, and 53/63 (84.1%) did not significantly change their MFR. (B) 2/23 (8.7%) cells significantly decreased their MFR when the arena shape was changed from square to circle, while 3/23 (13%) cells significantly increased their MFR, and 18/23 (78.3%) did not significantly change their MFR. (C) 2/9 (22.2%) cells significantly decreased their MFR when the arena was expanded, while 1/9 (11.1%) cells significantly increased their MFR, and 6/9 (66.7%) did not significantly change their MFR. (D) 4/15 (26.7%) cells significantly decreased their MFR when the arena walls were removed, and 11/15 (73.3%) did not significantly change their MFR.

Given that individual RSC neurons exhibited systematic mean firing rate changes that reflected the presence or absence of an object, we next wondered whether there was sufficient information at the population level to determine object condition. Accordingly, we conducted a population vector correlation analysis to compare temporally-matched mean firing rate population vectors, grouped across animals and sessions, between baseline and object conditions. A whole-session mean firing rate population vector was calculated using the mean firing rate of the whole session (765 s) consisting of all 200 cells. Next, a vector of all 200 cells was made by splitting the session into 400 millisecond (ms) bins and calculating the MFR separately for each bin (1912 bins). The whole-session population MFR vector for each session type (Pre-object, Object, Post-object) was then correlated with each 400 ms bin for the session of the same type and the other two session types (population MFR Pre-object vector correlated with binned Pre-object vector, population MFR Pre-object vector correlated with binned Object vector …). The correlations between the whole-session population vector and the binned MFR vector for sessions of the same type (e.g., Pre-object population & binned Pre-object) were more correlated than correlations between the population vector and the binned MFR vector of different sessions. However, all correlation distributions were significantly different from each other (Figure 4; Kruskal-Wallis, χ^2^ = 9559.3, P = 0, post hoc Scheffe test for multiple comparisons, P = 0). This latter result suggests that there is a change over long time intervals in the representation by the population of neurons. Note that the population change in Figure 4 must be because of changes over time in a separate group of neurons from those that show the mean firing rate changes in Figures 2 and 5, because the analysis of mean firing rate changes only selected neurons that maintained systematic changes in mean firing rate during the manipulation condition and therefore did not differ between the pre and post conditions.

### Manipulation of environmental features induces a change in mean firing rate

Characterization of RSC firing properties in the object manipulations were conducted with baseline sessions in which the open arena remained in a fixed location relative to the experimental room and fixed distal cues therein. In a separate set of experiments, we also found similar changes in mean firing rate when we conducted a series of experimental manipulations of the relationship between the familiar arena and the testing room. This allowed us to determine effects on mean firing rate changes of the current configuration of the boundaries of the arena and their relationship to the distal cues.

To examine what differences in the environment could produce an alteration to mean firing rate, the orientation of the environment with respect to the distal cues of the room was changed. In the manipulation session, we rotated the open field 45° to maximally disrupt correspondence between arena walls and distal walls or cues present within the recording environment from the pre-manipulation and post-manipulation sessions. This allowed us to study MFR changes that occurred due to changes in the relationship between the boundaries of the local arena and the cues and boundaries of the broader recording room. Under these conditions, we recorded a total of 63 RSC neurons of which 15.9% exhibited MFR changes (Figure 5A, n = 10/63). In 80% of these neurons, the mean firing rate significantly decreased in the object session compared to the pre-rotation and post-rotation sessions (n = 8/10). Conversely, 20% of these neurons, the mean firing rate significantly increased in the rotation session compared to the pre- and post-rotation sessions (n = 2/10).

To further examine whether differences in environment shape could produce a change in mean firing rate, we next manipulated the dimensions or geometry of the experimental arena. The arena was expanded in one direction, resulting in a rectangular arena, or the recording was performed in a circular arena in the middle session. The mean firing rate significantly changed in 25% of neurons recorded in all three sessions (Figures 5B and 5C, n = 8/32). In 50% of these neurons, the mean firing rate significantly increased in the manipulation session compared to the pre- and post-sessions in the square chamber (n = 4/8). Conversely, in 50% of these neurons, the mean firing rate significantly decreased in the manipulation session compared to the pre- and post-sessions in the square chamber (n = 4/8). To determine the effect of the presence of visual boundaries on the mean firing rate, we removed the walls of the arena in the middle session. In the no walls manipulations, 26.7% cells showed a significant increase in MFR (Figure 5D, n = 4/15). Together, these systematic changes in MFR demonstrate a signal of an alteration of the local environment, or the relationship between local and distal features of the environment.

## Discussion

Retrosplenial neurons display spatial correlates that are reliable within and across sessions and have diverse spatial responses. In the data presented here, a subset of RSC neurons exhibited reliable increases or decreases in mean firing rate when an object was inserted into the environment (Figure 2). Similar changes in mean firing rate occurred during large-scale changes to the environment such as changes in environment geometry, size, and orientation, or the presence of walls (Figure 5). These general changes in firing rate are consistent with previous broad changes in firing rate in the retrosplenial cortex associated with changes in the color of the environmental barriers, auditory, and olfactory stimuli (Miller et al., 2021). These changes in mean firing rate suggest a general, environment-spanning code for the presence of an object, or a general environment-spanning awareness of a change in the environment. A broad response to the presence of an object or a change in environment could reflect a type of working memory for the presence of a change that does not depend upon the animal's current location.

Changing the geometry of the arena, removing boundaries, or rotating the arena all produced a change in MFR in a subset of cells, indicating that these cells may be responding to a disruption in the relationship between the distal cues of the recording room and the local cues of the arena that the animal traverses. If the allocentric map is anchored to the relationship of local boundaries and landmarks to distal cues, this may explain the change in MFR. Retrosplenial cortex gets input from many regions, including visual cortex, hippocampal, and parahippocampal regions (Sugar et al., 2011). This convergence of sensory input and internal representations could allow an interaction of sensory information about distal and local cues with the internal allocentric representations from hippocampus and entorhinal cortex.

As noted above, the retrosplenial cortex shows changes in fMRI activation associated with coding the memory of landmarks within environments (Chrastil et al., 2015; Epstein et al., 2007; Marchette et al., 2015; Sherrill et al., 2013). The current electrophysiological data on insertion of objects are consistent with the interpretation that fMRI changes are associated with coding of objects by neural activity in this region. The cells that go back to their baseline MFR after the object has been removed or the arena has returned to the pre-manipulation indicate a configuration of neural activity that is influenced by visual information or landmark stability. This is consistent with a role of retrosplenial cortex in the learning of landmark stability in fMRI tasks (Auger et al., 2012, 2017; Patai et al., 2019).

The changes in mean firing rate observed here and in previous work (Miller et al., 2021) suggest a broad based code for changes in the environment that differ from the sharper tuning curves of place cells or grid cells responding more specifically to location, and the tuning curves of object-vector cells (Høydal et al., 2019) or landmark-vector cells responding at a specific distance and direction from objects (Deshmukh and Knierim, 2013). The neurons showing broad changes in mean firing rate were often accompanied in the same recordings by neurons that exhibited much more detailed tuning responses, such as egocentric boundary cells, head direction cells, and cells responding to running speed (Alexander et al., 2020; Jacob et al., 2017). This is the case in the current work, despite these spatial cell phenotypes being separate populations from the cells that systematically changed their mean firing rate after object insertion.

Population vector MFR correlations indicate a gradual change of overall firing over long time intervals in addition to the neurons that exhibit mean firing rate changes. Evidence for a general change in firing representations has been shown in the hippocampus during calcium imaging of place cells over days (Levy et al., 2021; Mau et al., 2018; Ziv et al., 2013) as well as during electrophysiological recording in region CA1 (Mankin et al., 2012). The shift in population correlation across sessions in our data could reflect this general temporal change in addition to the specific changes in mean firing rate associated with the object and environment manipulations. The temporal change continues as the environmental features change, despite a subpopulation of neurons also maintaining systematic changes in mean firing rate. This is shown by the gradual change in correlation, with the post-manipulation correlation distribution being the most different from the pre-manipulation distribution. This slower temporal change is happening while a subset of neurons are maintaining their systematic changes in MFR, potentially signaling the alteration in the environment as well, but returning to a baseline MFR when the environment returns to its previous configuration. The mean firing rate change induced by object insertion or environmental changes resembles the change in mean firing rate associated with change in running speed found in structures including the medial entorhinal cortex (Hinman et al., 2016; Kropff et al., 2015) and the primary visual cortex (Niell and Stryker, 2010). However, the small percentage of neurons showing changes in mean firing rate as well as tuning to running speed indicate that the mean firing rate changes found here were not because of an overall change in running speed in the different conditions.

As noted above, the change in mean firing rate to changes in environment orientation or size resembles the response to changes in environmental context in the form of barrier color, auditory, and olfactory cues, as shown in some neurons in the retrosplenial cortex (Miller et al., 2021). This effect was previously characterized as indicating the coding of spatial context in the retrosplenial cortex (Miller et al., 2021). Many previous studies have analyzed coding of the behavioral context of task requirements in other structures (Anderson and Jeffery, 2003; Jeffery et al., 2004). For example, neurons in hippocampus have been shown to change their responses based on tasks in which differences in spatial context required a different behavioral response to odor cups (Komorowski et al., 2009). However, the evidence of the changes in response in this previous study focused on context-dependent changes in a specific item response. Neuronal responses became more specific for conjunctions of an item in a specific spatial location, rather than exhibiting a global change in firing rate. Similarly, responses to spatial locations have also been shown to be modulated by the context of prior or future behavioral response in recordings from the hippocampus (Wood et al., 2000; Ferbinteanu and Shapiro, 2003; Bower et al., 2005) and from entorhinal cortex (Frank et al., 2000). The current work is notably different in that there was no task associated with interaction of the object and the object was not rewarded. The responses to environmental context differ in comprising a broad change in mean firing rate, rather than a change in tuning to specific items or places. The broadly distributed change in mean firing rate observed in the set of retrosplenial neurons presented here could provide a general signal to alter processing on a broad scale based on the presence of a general change in environment that influences the current internal environmental context maintained by the animal.

### Limitations of the study

Further work is important for understanding the mechanisms for coding of these different environmental changes and how it affects downstream targets, as well as which input regions are impacting the firing of these neurons in RSC, to determine the role RSC plays in scene processing and orientation within an environment. In addition, characterization of the different stimuli that produce this signal of environmental change are interesting next steps, such as an object being rewarded or involved in decision making, or if cells can code for multiple sensory stimuli dimensions.

## STAR★Methods

### Key resources table

**Table:** 

REAGENT or RESOURCE	SOURCE	IDENTIFIER
**Experimental models: Organisms/strains**		

Long-Evans rats	Charles River Laboratories	Crl:LE

**Software and algorithms**		

MATLAB version R2021a	The Mathworks, Inc.	https://www.mathworks.com/downloads/
Offline Sorter v3.3.6.1	PLEXON	https://plexon.com/products/offline-sorter/
DeepLabCut	Mathis et al., 2018	https://github.com/DeepLabCut/DeepLabCut

**Other**		

Neuralynx Digital Lynx SX	Neuralynx, Inc.	https://neuralynx.com/hardware/digital-lynx-sx
Leica CM3050S	Leica Biosystems	Configuration 1 (14903050S01)
Open Ephys Pellet Dispenser	https://github.com/jackpkenn/PelletDispenser	n/a
12μm nickel chromium tetrode wire	Kanthal-Sandvik	PF4803 RO800

### Resource availability

#### Lead contact

Additional information and resource requests should be directed to the Lead Contact, Lucas C. Carstensen (lucasc@bu.edu).

#### Materials availability

This study did not generate new unique reagents or materials.

### Experimental model and subject details

Adult male and female Long-Evans rats age 3-8 months (Charles River Labs, Wilmington, MA) were housed individually in plexiglass cages and kept on a 12-h light/dark cycle. Rats had continuous access to food during a habituation period lasting approximately 1 week. After this period, animals were food restricted until they reached 85-95% of their weight during free feeding. Water was available continuously. All procedures were approved by the Institutional Animal Care and Use Committee at Boston University.

### Method details

#### Shaping/behavior

Animals were acclimated to the primary testing room for approximately one week. During acclimation, rats were handled by multiple researchers and trained to consume both Froot Loops (General Mills, Battle Creek, MI) and 45mg chocolate pellets (Bio-Serv, Flemington, NJ). After animals readily ate both food items they were exposed to a familiar open fields used for baseline sessions for 12 to 35 minutes per day. The open field was 1.25m^2^ with 3 black walls and one white wall 30 cm in height. The arena was placed on a dark gray textured rubber floor that was cleaned between sessions.

#### Surgical procedures

Rats were surgically implanted with custom-fabricated hyperdrives in aseptic conditions. Each hyperdrive was composed of 12 to 16 nickel chromium tetrodes (12μm, Kanthal-Sandvik, Hallstahammar, Sweden) that could be independently moved in as small as 35μm increments. Guide cannula for each tetrode were collectively configured in one of three arrays: 1) filling a single hypodermic tube approximately 2mm^2^ in diameter, 2) across two conjoined hypodermic tubes that were ∼1.5mm^2^ in diameter spanning a total of ∼3mm or, 3) across four conjoined hypodermic tubes that were ∼1.25mm^2^ in diameter and spanned a total of ∼5mm. For the second and third configurations, the long axis of the electrode array was positioned to target an extended region of the anterior-posterior axis of retrosplenial cortex.

Animals were anesthetized using a combination of inhaled isoflurane (0.5% initial concentration) and ketamine/xylazine administered via intraperitoneal injection (Ketamine: 12.92 mg/kg, Acepromazine: 0.1mg/kg, Xylazine: 1.31 mg/kg). After the animal was determined to be under anesthesia (as assessed via loss of the toe pinch reflex), the animal was positioned in a stereotaxic apparatus, a 0.1mg/kg dose of atropine was administered subcutaneously, and the head was shaved. Excess hair was removed via application of Nair (Church & Dwight Co., Ewing, NJ) and the scalp was cleaned with 70% ethanol and Betadine (Avrio Health L.P., Stamford, CT). 0.9% sodium chloride was administered subcutaneously hourly throughout the surgical procedure.

Following a midline incision and subsequent clearing of connective tissue, a ground screw was positioned above the cerebellum and 5-8 anchor screws were affixed in an array around the perimeter of the exposed skull. A large craniotomy was centered above retrosplenial cortex (relative to bregma: A/P: -2mm to –7mm; M/L ±0mm-1.75mm). The exact size and position of the craniotomy was dependent upon the aforementioned configuration of the hyperdrive array. Next, dura was resected and the hyperdrive was positioned such that guide cannula rested gently against the dorsal surface of the brain. Excess exposed tissue within the craniotomy was protected with Kwik-Sil (World Precision Instruments, Sarasota, FL), and the implant was secured to anchor screws with dental cement. Tissue around the implant was cleaned with saline, 70% ethanol, and hydrogen peroxide. Antibiotic ointment was applied into the wound, sutured if necessary, and Neosporin was applied around the site. Prior to removal from anesthesia tetrodes were lowered approximately 0.25mm D/V. Animals received post-operative antibiotics (Baytril: 10mg/kg) and analgesics (Ketofen: 5.0mg/kg) for five days after surgery and were freely fed. After one-week post-operation animals were handled and re-acclimated to the testing room and free foraging environments prior to the initiation of experiments.

#### Electrophysiological recordings

Neural signals were amplified at two headstages attached to a 64 channel electrical interface board and acquired by a 64 channel Digital Lynx SX acquisition system (Neuralynx, Bozeman, MT) Signals were digitized, filtered (0.3-6.0kHz), and amplified (5,000-20,000X). Timestamps of individual action potentials were detected online when the signal crossed an acquisition threshold on any individual electrode composing a tetrode. At the conclusion of each experiment, spikes were manually sorted to individual single units using Offline Sorter (Plexon Inc., Dallas, TX) and the following features: peak-valley, peak, and principal components 1-3. Two diodes attached to the electrode implant delineated the location of the animal which was tracked at 30Hz via a camera positioned above the recording arena. Position estimates were generated using DeepLabCut (Mathis et al., 2018).

An experimental session began with an initial 12-35 minute recording while the animal free explored in a 1.25m^2^ arena and consumed either Froot Loops or chocolate pellets scattered by an experimenter, chocolate pellets released at random intervals from a dispenser positioned above the arena, or both (Open Ephys Pellet Dispenser designed by Maurer Lab, https://github.com/jackpkenn/PelletDispenser). After adequate spatial coverage was achieved the animal was removed from the arena and placed back in its home cage for a period of 1 hour minimally. Next, a second experimental session was conducted in which the animal explored an open field in one or more of the following configurations:

1 .Open field object session: The same arena from the baseline session to assess the firing of RSC neurons when an object is inserted into the environment. In experimental sessions, the object was a red, blue, or yellow Lego tower tall enough that the rat could not walk over it (7x7x26cm). The object was not novel to the animal for any sessions included in analyses. 200 cells were recorded in 3 rats in object manipulations.
2 .Open field rotation: The same arena from the baseline session rotated 45° relative to the testing room and all visible distal cues present therein. 63 cells were recorded in 3 rats in arena rotation manipulations.
3 .Circular open field: A familiar circular arena of 1.1m diameter. 23 cells were recorded in 2 rats in circular open field manipulations.
4 .Open field expansion or contraction: If an expansion experiment was planned the initial baseline session was conducted in a familiar 1.25m^2^ arena that enabled reconfiguration of walls. Following the baseline session, walls were uniformly moved outwards relative to the center point of the baseline configuration to a size of 1.5m^2^ or larger. 9 cells were recorded in 3 rats in expansion or contraction manipulations.
5 .No walls session: Walls were removed from the open field arena which was suspended approximately 60 cm above the floor so the animal would be discouraged from jumping off of the arena. The dimensions remained the same as in baseline open field sessions. 15 cells were recorded in 2 rats in wall removal manipulations.

After the manipulation session, the animal was returned to its home cage and the arena was returned to the same configuration as the pre-manipulation session. After period of 1 hour minimally, the animal was returned to the arena and allowed to forage for approximately 20 minutes. This A-B-A’ paradigm was followed for all environmental manipulations, with only the middle “B” session changing. The duration of the shortest experimental recording session was 765 seconds, so all analyses were performed on the beginning 765 seconds of each recording only. All arenas were positioned such that the animal could easily see the broader recording room and an array of stable distal cues. All three sessions were performed on the same day.

#### Histology

Animals were anesthetized with 0.5% isoflurane and small electrical lesions were made at the end of tetrodes on which cells had been recorded. After one week, animals were deeply anesthetized with isoflurane, injected with sodium pentobarbital, and transcardially-perfused with 0.9% saline followed by 10% formalin. The brain was extracted from the skull and post-fixed overnight with 10% formalin, then stored in 0.1M phosphate buffer until two days before slicing when it was transferred to a 0.1M phosphate buffer/30% sucrose solution. The brain was snap frozen using 2-methylbutane and sliced into 40-50um coronal sections using a cryostat (Leica CM3050S, Leica Biosystems, Buffalo Grove, IL). Slices were mounted on gelatin covered microscope slides and allowed to dry, then photographed (Nikon DS-Fi3 microscope camera mounted on Olympus BX51 light microscope). Tetrode tracts were clearly visible in all animals. Coordinates of tetrode locations and final tetrode depths were registered with respect to pre-implant photographs of guide cannula array configurations and tetrode turning logs, respectively. Histology for cells recorded in environmental manipulation sessions is included in Alexander et al., 2020. All cells that could be verified as in RSC from histology and turning logs were considered RSC neurons. Most tetrodes passed through dysgranular RSC and tetrodes were balanced across hemispheres. All cells included in analyses were required to be present in all 3 sessions, the manipulation session only, or the pre- and post-manipulation sessions only. Inhibitory cells were not excluded.

### Quantification and statistical analysis

#### Data analysis

##### 2-Dimensional spatial ratemaps

Animal positional occupancy within an open field was discretized into 3 cm × 3 cm spatial bins. For each neuron, the raw firing rate for each spatial bin was calculated by dividing the number of spikes that occurred in a given bin by the amount of time the animal occupied that bin. Raw firing ratemaps were smoothed with a 2D Gaussian kernel spanning 7 cm to generate final ratemaps for visualization. Pixels that were not visited were not included in analyses.

##### Linear speed tuning

Linear speed tuning curves were constructed for all neurons for each session using 25 bins spanning speeds ranging from 5 to 55cm/s (2 cm/s bins). Each tuning curve for each neuron was fit with a uniform and linear function. F-tests were run to compare the residuals of these two fits. Neurons with p<0.05 for this comparison in either the pre-object, object, or post-object session were determined to possess linear speed sensitivity.

#### Statistics

Normality of distributions was not assumed. Nonparametric tests with a P value threshold at 0.05 were used for all statistical comparisons. Kruskal-Wallis tests to determine whether samples originated from the same distribution were used to compare mean firing rate changes between sessions and for comparison of population vector correlation distributions. Resulting statistics from the Kruskal-Wallis test were used for post-hoc multiple comparisons tests. All statistical tests were conducted using MATLAB computing software.

## Data Availability

• Data will be made available by the lead contact upon request. • Custom MATLAB code will be made available by the lead contact upon request. • Any additional information required to reanalyze the data reported in this paper is available from the lead contact upon request.
