# Supplementary material for: Neural responses in retrosplenial cortex associated with environmental alterations
Source: iScience. 2021 Oct 28;24(11):103377. doi: 10.1016/j.isci.2021.103377 (PMC8605176; doi:10.1016/j.isci.2021.103377)
Supplement: Document S1. Figures S1 and S2 [file mmc1.pdf]

iScience, Volume *24*

Supplemental information

Neural responses in retrosplenial cortex associated with environmental alterations

Lucas C. Carstensen, Andrew S. Alexander, G. William Chapman, Aubrey J. Lee, and Michael E. Hasselmo

Supplemental Information

<u>Supplemental Figures and Legends</u>

Supplemental Figure 1

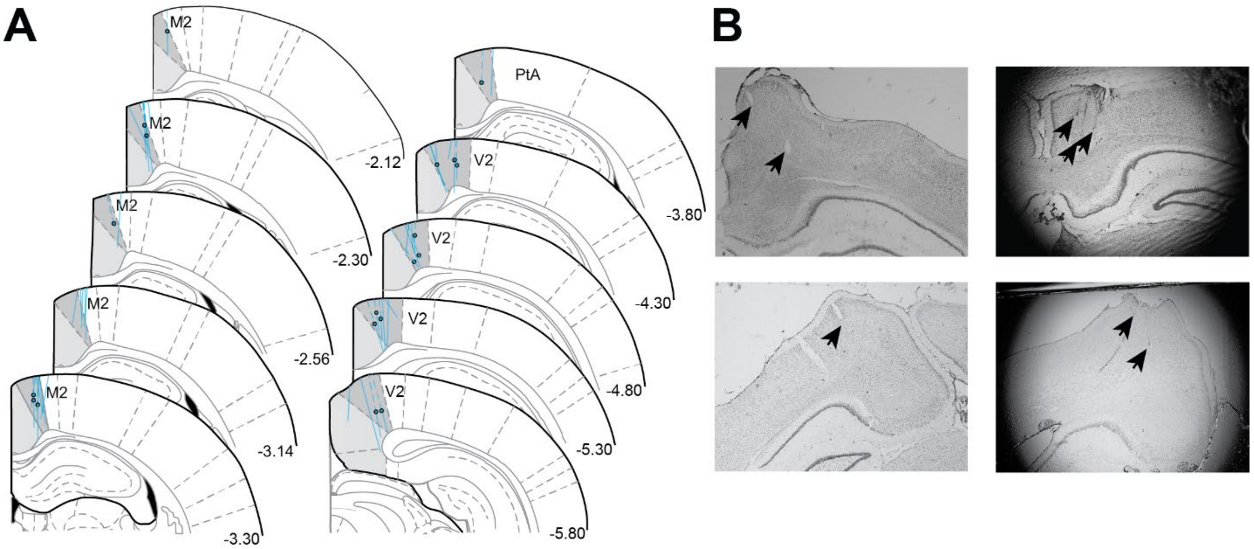

Figure S1. Locations of cells with significant changes in mean firing rate, Related to Figure 1. (A) Dashed lines indicate locations of retrosplenial cortex (RSC) tetrode tracts where neurons with significant changes in MFR were observed. Circles indicate the most ventral location in which a neuron with a significant change in MFR was recorded. Dark gray indicates dysgranular RSC and light gray indicates granular RSC. **(B)** Example histology showing tetrode locations in RSC. Tetrode locations are marked with black arrows.

Supplemental Figure 2

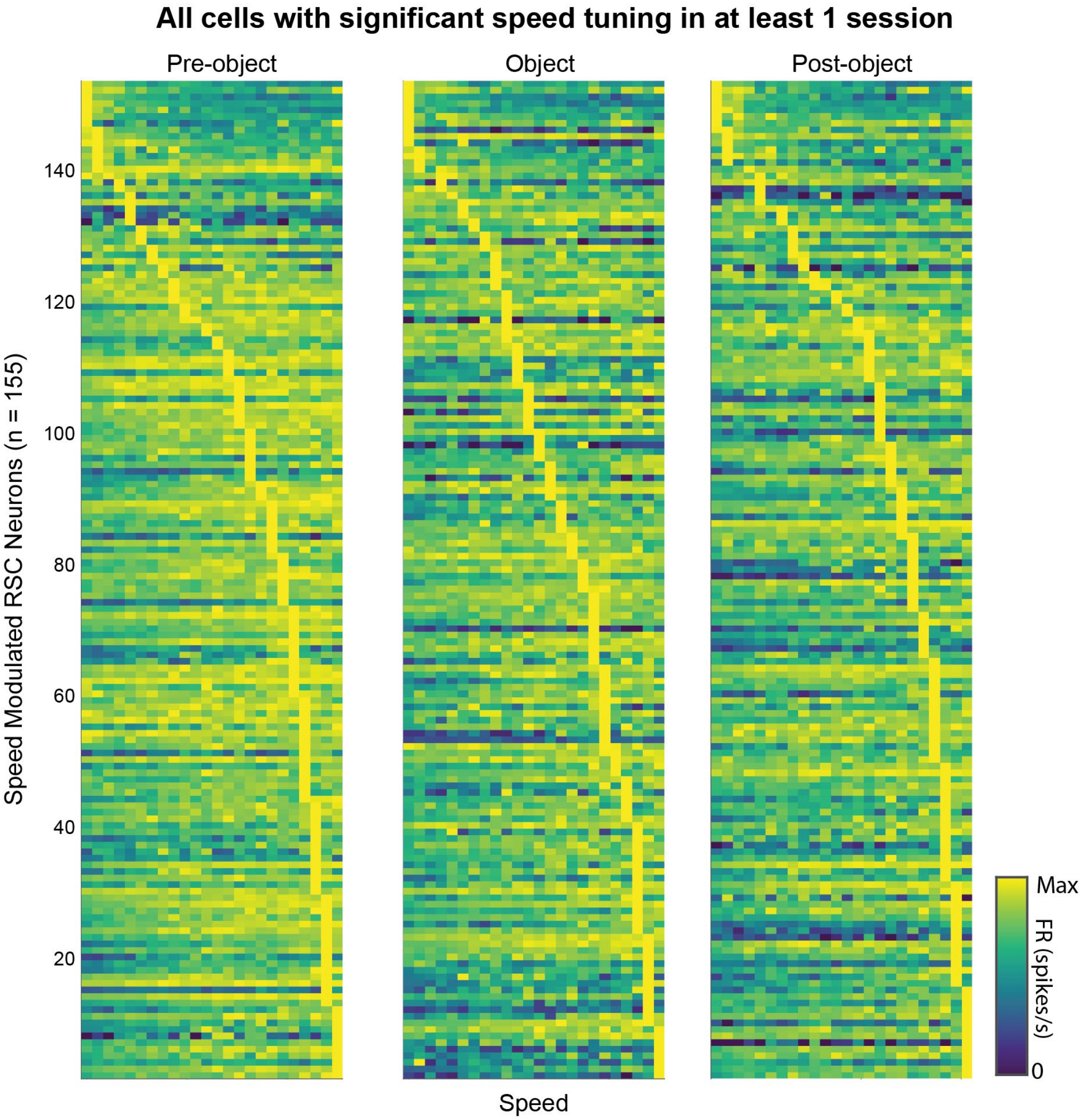

Figure S2. Speed modulation of retrosplenial neurons split by session, Related to Figure 3. Retrosplenial cells show a spectrum of speed modulation responses. Each row shows the speed modulated firing rate across speed bins from 0 to 60 cm/sec in bins of 2.5 cm/sec. Each session is shown separately. Cells were sorted by the speed at which their modulated firing rate was highest for each session.
